# Design and Implementation of Cloud Analytics-Assisted Smart Power Meters Considering Advanced Artificial Intelligence as Edge Analytics in Demand-Side Management for Smart Homes

**DOI:** 10.3390/s19092047

**Published:** 2019-05-02

**Authors:** Yung-Yao Chen, Yu-Hsiu Lin, Chia-Ching Kung, Ming-Han Chung, I-Hsuan Yen

**Affiliations:** 1 Graduate Institute of Automation Technology, National Taipei University of Technology, Taipei 106, Taiwan; yungyaochen@mail.ntut.edu.tw; 2 Department of Electrical Engineering, Allied AI Biomedical Research Center, Southern Taiwan University of Science and Technology, Tainan 71005, Taiwan; 4a52c007@stust.edu.tw (C.-C.K.); 4a52c032@stust.edu.tw (M.-H.C.); 4a52c010@stust.edu.tw (I.-H.Y.)

**Keywords:** artificial intelligence, cloud analytics, demand-side management, edge/fog analytics, electrical energy management, Internet of things, smart grid, smart homes/factories, smart sensing

## Abstract

In a smart home linked to a smart grid (SG), demand-side management (DSM) has the potential to reduce electricity costs and carbon/chlorofluorocarbon emissions, which are associated with electricity used in today’s modern society. To meet continuously increasing electrical energy demands requested from downstream sectors in an SG, energy management systems (EMS), developed with paradigms of artificial intelligence (AI) across Internet of things (IoT) and conducted in fields of interest, monitor, manage, and analyze industrial, commercial, and residential electrical appliances efficiently in response to demand response (DR) signals as DSM. Usually, a DSM service provided by utilities for consumers in an SG is based on cloud-centered data science analytics. However, such cloud-centered data science analytics service involved for DSM is mostly far away from on-site IoT end devices, such as DR switches/power meters/smart meters, which is usually unacceptable for latency-sensitive user-centric IoT applications in DSM. This implies that, for instance, IoT end devices deployed on-site for latency-sensitive user-centric IoT applications in DSM should be aware of immediately analytical, interpretable, and real-time actionable data insights processed on and identified by IoT end devices at IoT sources. Therefore, this work designs and implements a smart edge analytics-empowered power meter prototype considering advanced AI in DSM for smart homes. The prototype in this work works in a cloud analytics-assisted electrical EMS architecture, which is designed and implemented as edge analytics in the architecture described and developed toward a next-generation smart sensing infrastructure for smart homes. Two different types of AI deployed on-site on the prototype are conducted for DSM and compared in this work. The experimentation reported in this work shows the architecture described with the prototype in this work is feasible and workable.

## 1. Introduction

There is a growing interest in applying recent breakthrough technologies in relevant fields such as smart homes in smart cities. Recent breakthrough technologies are trending for today’s technologically driven society, from the fundamental constituents of a city, smart homes, buildings, and factories in the Fourth Industrial Revolution (Industry 4.0), to smart cities. Smart cities are derived from the intense deployment of Internet of things (IoT) technologies with artificial intelligence (AI). The technical combination of IoT technologies with AI brings novel insights into home automation, home healthcare, home surveillance, and home energy management in smart homes as examples in today’s modern society.

Electrical energy forms an indispensable part of today’s modern society; people’s lives would be impossible without the aid of electricity, which is one of the most common and important commodities in use every day. To meet continuously increasing electricity energy demands requested from downstream sectors of a smart grid (SG) and reduce greenhouse gases such as carbon dioxide and chlorofluorocarbons produced, it is of utmost importance to monitor and manage industrial, commercial, and residential electrical appliances. The traditional power grid/electrical power system expected to monitor and manage electrical appliances effectively in electrical energy management is inadequate to overcome modern-day challenges addressed in an SG [1]. An SG that incorporates ICT (information and communication technologies)/IoT with AI innovatively appears as the next generation of the traditional power grid [2]. In an SG, one of the most important functionalities developed by utilities and provided for consumers is the self-decision-making ability. To enable the self-decision-making ability in an SG, utilities developed viable and effective demand response (RD) mechanisms for demand-side management (DSM)/electrical energy management [3,4,5,6]. In an SG, DSM refers to initiatives and technologies that encourage end customers/consumers to optimize their electrical energy consumption patterns in response to DR signals, while improving the stability and reliability of the compromised traditional power grid that serves as an SG. To participate in DR programs/DSM in an SG, the first important step is to keep track of fine-grained electrical energy consumed by individual major electrical appliances in practical fields of interest. For example, in a residential field linked to an SG via advanced metering infrastructure (AMI), a smart home energy management system (EMS) can be conducted for DSM/electrical energy management [1,3,4,5,6] and installed with an intrusive deployment of smart plugs [3,4,7,8] that connect with a power company-owned smart meter in AMI and keep track of electric power consumption on each individual major electrical appliance monitored. The worldwide adoption of EMSs that identify and communicate electrical energy consumption data with an intrusive deployment of smart plugs [3,4,7,8] gives rise to new user-centric IoT applications, electrical energy efficiency services, in DSM. However, it is based on a centralized cloud architectural model [9,10]; in smart homes connected with a smart city in an SG, deployed IoT end devices generate substantial amounts of data that must be transmitted, stored, and processed in powerful cloud computing. Cloud-centered data science analytics, cloud analytics, is based on network connectivity/the internet, which is not always available or is limited, which is usually unacceptable for latency-sensitive user-centric IoT applications developed for smart home services in an SG [11]. Compared with cloud analytics, where data gathered by IoT end devices and transmitted to cloud storage are treated in centralized cloud-centered data science analytics, edge analytics (fog computing), a promising technique dedicated and used to analyze data that need to be processed for immediately analytical, interpretable, and real-time actionable data insights at IoT sources, extends cloud analytics (to the edge of the internet) and covers its shortage. As a result, edge analytics that supports immediately analytical, interpretable, and real-time actionable data insights at IoT sources is necessary for latency-sensitive user-centric IoT applications in smart homes, where AI models are trained in cloud analytics and then deployed on-site on IoT end devices as edge analytics at the edge of the internet. In this sense, fog-cloud analytics is formed [12,13].

In fog-cloud analytics, IoT end devices, edge devices or edge sensors, can be gateways, industrial controllers/switches, routers, and video surveillance cameras/vision sensors in smart homes, manufacturing, and cities considering DSM, and they can process data on-site for immediately analytical, interpretable, and real-time actionable data insights and store data near where data processed are produced on-site for latency-sensitive user-centric IoT applications. In DSM, fog-cloud analytics developed and conducted for latency-sensitive user-centric IoT applications can serve as converged analytics that consolidates data from distributed data aggregation as a next-generation AMI/smart sensing infrastructure. For example, power meters can be distributed and deployed in multiple fields of interest for DSM in an SG. Each deployed power meter is used to gather micro data transmitted to and analyzed in cloud analytics. AI trained in cloud analytics and converged into resulting macro insights is then deployed on-site/sent back to each deployed power meter to perform edge analytics in fields of interest.

As motivated above, edge analytics is necessary for latency-sensitive user-centric IoT applications in DSM, where immediately analytical, interpretable, and real-time actionable data insights processed on and identified by IoT end devices at IoT sources are required and gained with cloud analytics collaborated together [11,14,15]. Also, much attention remains to be paid in conducting and applying fog-cloud analytics in smart homes, as examples, for DSM/electrical energy management in an SG. Therefore, this work designs and implements a smart edge analytics-empowered power meter prototype considering AI in smart homes for DSM/electrical energy management. The prototype in this work works in a cloud analytics-assisted electrical EMS architecture, which aims to bring AI trained in cloud analytics and then embedded/deployed on-site on the designed and implemented prototype as edge analytics. The AI-embedded smart power meter prototype designed and implemented in this work is based on an Arduino micro-controller unit (MCU). Arduino is an open-source electronics prototype MCU based on flexible and easy-to-use hardware and software, which is very popular amongst artists, designers, hobbyists, and professionals. Arduino MCU is an excellent tool, which can be used to quickly test and prototype ideas. The specification of load signature, which is used in load monitoring as load identification, without an intrusive deployment of smart plugs for electrical appliances in fields of interest, is another important aspect in DSM, as identifying electrical appliances via an intrusive deployment of smart plugs installed for monitored electrical appliances is laborious and cost-intensive [3,7,8,16,17,18,19,20]. Thus, a load monitoring approach, the designed and implemented AI-enabled smart power meter prototype presented as edge analytics and collaborated together with cloud analytics, is developed in fog-cloud analytics in this work. The load monitoring approach identifies electrical appliances with no intrusive deployment of smart plugs installed for electrical appliances monitored in the architecture described. AI, such as artificial neural networks (ANNs) used to perform (on-line) load monitoring in this work, analyzes collected data at sensor points rather than waiting for data collected and transmitted to a cloud/on-premise server for further data analysis. In this work, two different types of ANN are conducted and compared. ANNs in AI are powerful connectionist systems vaguely inspired by biological neural networks. The widely used backpropagation ANN (BP-ANN) [8,16,17,18,19,21] and radial basis function ANN (RBF-ANN) incorporated with fuzzy C-means (FCM) clustering are considered and compared in this work. The superior AI model trained in cloud analytics and compared is then deployed on-site on the Arduino MCU-based smart power meter prototype as edge analytics in the architecture described in this work. For the push notification service, IFTTT (if this, then that) is implemented, in the described architecture, with LINE Notify. A proof-of-concept demonstration reported in this work experimentally confirms that the architecture described with the prototype in this work is feasible and workable. The architecture described in this work and presented with the designed and implemented AI-embedded smart power meter prototype is a preliminary design toward a next-generation AMI/smart sensing infrastructure motivated previously and dedicated for advances in smart homes, manufacturing, and cities considering DSM. The work done in this work is an extended effort against the work that was finished in References [21,22,23], which is also a comparative study done with References [21,23]. The prototype designed and implemented in the described architecture in this work can be further developed, through preventive analysis, for preventative maintenance in DSM.

The remainder of this work is structured as described below. Section 2 shows a brief overview of related work done in DSM/electrical energy management. The cloud analytics-assisted electrical EMS architecture collaborated together with the designed and implemented Arduino MCU-based smart power meter prototype as edge analytics for DSM is described in Section 3. Improved AI, the FCM clustering/piloting RBF-ANN compared with the BP-ANN in References [21,23] and used by the AI-enabled smart power meter prototype in this work, is also described in Section 3. In Section 4, a proof-of-concept demonstration of the described architecture with the smart prototype in this work is shown. Lastly, Section 5 concludes this work with its future work.

## 2. Related Work

Significant research, in recent years, was carried out to identify and communicate electrical energy consumption data with an intrusive deployment of smart plugs based on EMSs for new user-centric IoT applications, electrical energy efficiency services, in DSM/electrical energy management.

In Reference [9], the authors proposed a self-learning home management system comprising (1) an EMS, (2) a DSM system, and (3) a supply-side management system. The three parts of the home management system were developed and integrated for the real-time operation of a smart home. The functionalities of the centralized and integrated home management system include price forecasting, price clustering, and power alert system capabilities for smart home energy management. In Reference [10], the authors aimed to develop a multilayer cloud architectural model that enables effective and seamless interactions/operations on heterogeneous IoT end devices from different IoT smart homes. In addition, an ontology-based security service framework was developed and used to support security and privacy preservation in the process of the interactions/operations of the heterogeneous IoT devices. The model developed in Reference [10] is a centralized cloud architectural model. In smart homes connected with a smart city in an SG, deployed IoT end devices generate substantial amounts of data. Substantial amounts of data generated from IoT end devices deployed in smart homes in a smart city in an SG must be transmitted, stored, and processed in powerful cloud computing, which is usually unacceptable for latency-sensitive user-centric IoT applications developed for consumers in smart homes in a smart city in an SG [11]. This is because powerful cloud computing, cloud-centered data science analytics/cloud analytics, is based on network connectivity/the internet, which is not always available or is limited. Compared with cloud analytics where data gathered by IoT end devices and transmitted to cloud storage are treated in centralized cloud-centered data science analytics, edge analytics/fog computing extends cloud analytics (to the edge of the internet) and covers its shortage. Edge analytics is a promising technique dedicated and used to analyze data that need to be processed for immediately analytical, interpretable, and real-time actionable data insights at IoT sources. As a result, edge analytics that supports immediately analytical, interpretable, and real-time actionable data insights at IoT sources is needed for latency-sensitive user-centric IoT applications in smart homes, where AI models are trained in cloud analytics and then deployed on-site on IoT end devices as edge analytics at the edge of the internet. In this sense, fog-cloud analytics is formed; resulting insights from cloud analytics are sent back to edge analytics. In Reference [12], the authors presented a simulated OpenFog reference architecture-based smart home system considering fog-cloud computing. However, the simulated system was not evaluated in a realistic environment; a practical evaluation was lacking in the study. Also, the study was absent in terms of a demonstration of analysis capabilities in which AI is trained in cloud analytics and deployed on-site on IoT end devices as edge analytics for user-centric IoT applications in smart homes. In Reference [11], the authors concentrated on a fog computing-based home automation system that allows for seamless communication among IoT end devices for heterogeneous communication technologies. Nevertheless, the research finished in Reference [11] was also absent in terms of a demonstration of analysis capabilities in that no AI is trained in cloud analytics and deployed on-site on the IoT end devices for IoT applications. The IoT end devices did not act as edge analytics in the research. The system developed in Reference [11] can be further developed for DSM in smart homes. In Reference [13], the authors studied a fog computing-based Internet of energy (IoE) architecture for transactive energy (TE) management systems. TE management utilizes optimal day-ahead energy consumption scheduling and an inter-customer energy trading mechanism for exchanging electrical energy among end users. A real testbed consisting of an IoT end device and hypertext transfer protocol (HTTP) gateway connected over the internet with a cloud server was implemented and used to experimentally evaluate the fog computing-based architecture in the study. However, no demonstration of on-site deployment of AI for IoT end devices as edge analytics in fields of interest was addressed in the study; IoT end devices implemented in the fog computing-based architecture/testbed in the study are not capable of non-intrusively identifying electrical appliances used in smart homes.

The work developed here against related work in the literature above is summarized as follows: firstly, a smart edge analytics-empowered power meter prototype considering AI in smart homes for DSM/electrical energy management in an SG is designed, implemented, and practically evaluated in this work, which works in a cloud analytics-assisted electrical EMS architecture. The architecture described in this work brings AI trained in cloud analytics and then embedded/deployed on-site on the designed and implemented power meter prototype as edge analytics. Secondly, a load monitoring approach, the designed and implemented AI-enabled smart power meter prototype collaborated together with cloud analytics and used as edge analytics to non-intrusively identify electrical appliances with no intrusive deployment of smart plugs installed for electrical appliances monitored in the described architecture, is developed in fog-cloud analytics in this work. In this work, AI, BP-ANN and RBF-ANN incorporated with FCM clustering, is investigated. Thirdly, a push notification service based on IFTTT with LINE Notify is considered in DSM and implemented in the described architecture.

## 3. Methodology

**Figure 1:**
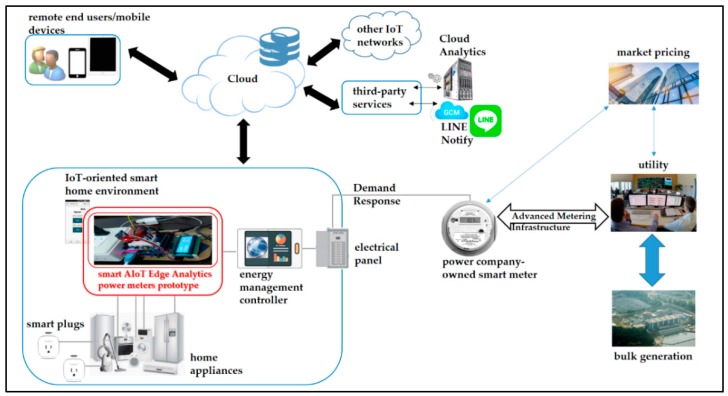
Typical home energy management system (EMS) model involving three main stakeholders.

The cloud analytics-assisted electrical EMS architecture collaborated together with the AI-enabled and Arduino MCU-based smart power meter prototype, smart AIoT (AI across IoT) edge analytics-empowered power meter prototype, with a push notification service for DSM in smart homes, is described in this section. Section 3.1 describes the cloud analytics-assisted electrical EMS architecture; Section 3.2 presents the AI (FCM clustering/piloting RBF-ANN)-enabled Arduino MCU-based smart power meter prototype designed and implemented, in the described architecture with push notification service, for edge analytics in this work. Home EMS is crucial for DSM in an SG, which can manage, control, and optimize electrical energy consumption in home environments. A home EMS serves as a bi-directional communication interface between a residential environment that responds to DR signals based on market pricing and an electric utility that monitors, controls, and analyzes gathered electrical energy consumption data from smart homes [24]. In this sense, involved communication technologies, wide area network (WAN), neighborhood area network (NAN), and home area network (HAN) [11,25,26,27], are the preliminaries used for DSM in smart homes connected in an SG. Figure 1 depicts a typical home EMS model. The typical home EMS model studied in this work is composed of (1) a power company-owned smart meter (instead of a power company-owned traditional wattmeter) communicated with a utility via AMI and used to transmit data records/electrical energy consumption data and receive DR signals, based on market pricing, for highly efficient generation of electricity by the utility from bulk generation; (2) a central energy management controller (EMC) installed in a smart home environment and communicated, via the internet, with a cloud, providing user-centric IoT applications for homeowners; and (3) home appliances monitored remotely and reacted with DR signals received.

In Figure 1, AMI referring to automated metering and advanced data management is responsible for measuring, collecting, and managing electrical energy consumption data from the smart meter to the utility. The smart meter acts as a communication gateway between the smart home environment and the utility, receiving electricity pricing signals/DR signals from the utility with market pricing for DSM in an SG. It is associated with electrical energy consumption data gathered from the EMC and transmitted to the utility via AMI for further data science analytics.

Further data science analytics in Figure 1 is based on cloud-centered data science analytics. Electrical energy consumption data transmitted to and electricity pricing signals received from the utility are delivered through the commonly available fixed networks, PLC (power line communication), GSM (global system for mobile communications), and/or WiMax [28,29].

In a typical home EMS model, it is assumed that each home environment is equipped with an EMC. An EMC installed is able to communicate with smart plugs intrusively attached to/deployed on electrical home appliances monitored for DSM in a home environment. Smart plugs can use ZigBee as the communication protocol [3,7,8]. An EMC installed also serves as an in-home display, to realize data visualization and remote load control in a home environment. Typically, an EMC is configured with heterogeneous communication protocols [11,30,31,32] in an HAN, and it is based on an ARM^®^ (Advanced (Reduced Instruction Set Computing) RISC Machine) processor-based embedded system [1,3,4,7,8].

**Figure 2:**
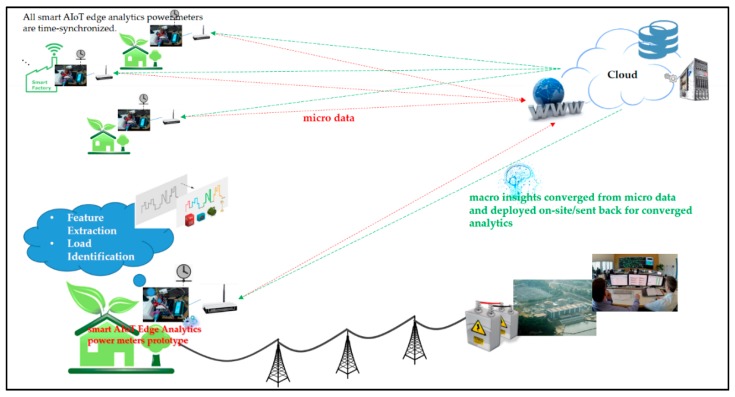
Conceptual vision of the presented prototype considering a future user-centric Internet of things (IoT) application, based on fog-cloud analytics, for demand-side management DSM in a smart grid (SG). Advances, user-centric IoT service-oriented single and/or multiple sensing modalities, for DSM in an SG and many others in a smart city can build upon this infrastructure.

In the typical home EMS model depicted in Figure 1, the smart AIoT edge analytics-empowered power meter prototype studied in this work, collaborated together with cloud analytics and presented later, is needed. This is because data science analytics for user-centric IoT applications in DSM in Figure 1 is based on (centralized) cloud analytics and a future fog-cloud analytics-enabled AMI/smart sensing infrastructure for DSM in an SG is expected. The conceptual vision of the future fog-cloud analytics-based AMI/smart sensing infrastructure expected is shown in Figure 2.

In Figure 2, the smart AIoT edge analytics-empowered power meter prototype, the AI-enabled and Arduino MCU-based smart power meter prototype, can be deployed in multiple fields of interest in an SG. All smart AIoT edge analytics power meters distributed and deployed for DSM in an SG are time-synchronized. Micro data gathered and analyzed by each of local smart AIoT edge analytics power meters are transmitted to cloud storage and converged in cloud analytics. Macro insights by AI trained in cloud analytics and then deployed on-site on each of local smart AIoT edge analytics power meters are sent back to IoT sources/end users. To deploy AI, which is trained in cloud analytics, on-site on the Arduino MCU-based smart power meter prototype, OTA (over the air) functionality [17] conducted in Figure 1 for edge analytics can be extremely useful in cases of limited or no physical access to MCUs that update their firmware remotely.

The architecture with the prototype considering the FCM-piloting RBF-ANN to perform on-line load monitoring in this work is described below, which is a preliminary design toward such a scenario in Figure 2.

### 3.1. Cloud Analytics-Assisted Electrical EMS Architecture

**Figure 3:**
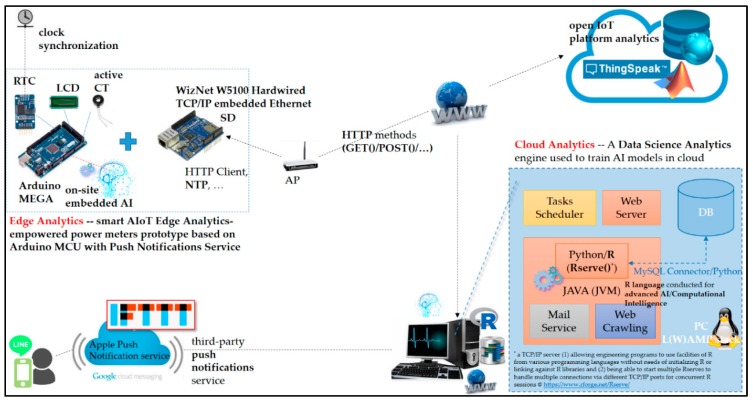
Described cloud analytics-assisted electrical EMS architecture empowered by the artificial intelligence (AI)-embedded and Arduino micro-controller unit (MCU)-based smart power meter prototype as edge analytics.

Figure 3 depicts the described cloud analytics-assisted EMS architecture that comes up with the IoT technology stack in References [21,22,23], using the typical home EMS model studied in Figure 1, and considering the AI (the FCM clustering-piloting RBF-ANN)-empowered and Arduino MCU-based smart power meter prototype as edge analytics for DSM in this work. The push notification service is also considered in the architecture. In Figure 3, a WampServer, (1) Apache^TM^ HTTP server (Apache Software Foundation, Forest Hill, MD, USA), (2) MySQL^TM^ relational database (Oracle Corporation, Redwood City, CA, USA), and (3) PHP hypertext preprocessor (PHP) scripting language (Rasmus Lerdorf, Qeqertarsuaq/Disko Island, Greenland) sous the Windows^®^ operating system (OS), is configured on the personal computer (PC)-based data science analytics engine as cloud analytics in this work. The PC serves as the core/central entity of the data science analytics engine as cloud analytics, where the WampServer is established with a mashup of Java^TM^, R language, and Python. The WampServer is a Windows web development environment, which allows developers to create web applications with an Apache HTTP server, MySQL^TM^ relational database, and PHP. Alongside, phpMyAdmin allows developers to easily manage their databases. A mashup of Java^TM^, R language, and Python is suited for data science analytics/AI trained in cloud analytics. The Java Virtual Machine configured and shown in Figure 3 is a virtual machine. It enables the configured data science analytics engine to run cross-platform Java programs, as well as programs coded in other programming languages, where programs are also compiled to Java bytecode. R language, supported by the R Foundation [33], is a free software environment for statistical computing and graphics; it publicly provides a free package repository that features more than 11,800 available software packages covering from “Machine Learning and Statistical Learning” to “Graphics” for data analysis and visualization. The representational state transfer (REST)-ful Web Services application programming interface (API) [21] is also suited for AI in cloud analytics in this work.

**Figure 4:**
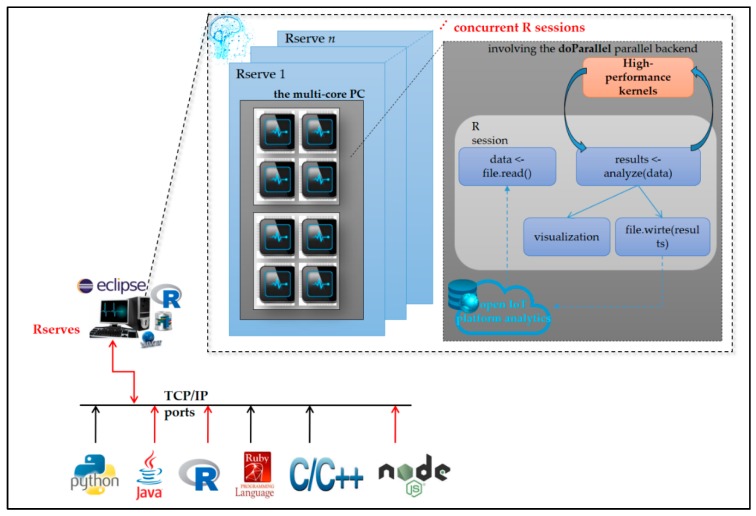
Rserve() in R language conducted and configured on the data science analytics engine for high-performance parallel processing/computing.

Figure 4 shows the Rserve() in R language, which is conducted and also configured on the data science analytics engine for high-performance parallel processing/computing, where, Rserve(), a Transmission Control Protocol/Internet Protocol (TCP/IP), socket server, (1) allows programs to use facilities of R from various programming languages without the need to initialize R or link programming languages against R libraries, and (2) is capable of starting multiple Rserves() to handle multiple connections via different TCP/IP ports for concurrent R sessions.

The software R packages “nnet” (applied for BP-ANN), “stats” (applied for k-means clustering), “fclust”/“e1071” (applied for FCM clustering), and “neural”/“RSNNS” (applied for RBF-ANN) provide feed-forward ANN algorithms and partitioning-based clustering algorithms hybridized and used in this work. The FCM clustering/piloting RBF-ANN trained in cloud analytics on the data science analytics engine and deployed on-site on the AI-enable and Arduino MCU-based smart power meter prototype as edge analytics is presented in the next section. High-performance parallel processing/computing in Rserve() can speed up ANN processes.

The widely used BP-ANN model and FCM clustering/piloting RBF-ANN model are trained in cloud analytics and compared in this work. Then, the superior AI model is embedded/deployed on-site on the presented smart Arduino MCU-based power meter prototype. The presented AI-enabled and Arduino MCU-based smart power meter prototype, used in a residential environment to monitor electrical appliances for DSM in an SG, is designed and implemented as edge analytics in the described architecture in Figure 2 and Figure 3. Fog-cloud analytics that involves collecting and analyzing sensor data at sensor points of the internet is considered in this work for advances in future user-centric IoT applications in Figure 2. In Figure 3, the Gmail Simple Mail Transfer Protocol (SMTP) e-mail service can be conducted as a third-party service in Figure 1, where the Highcharts Serverside Export framework providing a Java API for Highcharts, including image generation capabilities for routine electrical energy reports, is suited. In this work, to the third-party push notification service in Figure 1 and Figure 3, IFTTT (if this, then that) is conducted and used in the described architecture to send LINE Notify messages. IFTTT is a real free and handy way to get all developers’ apps and devices talking to each other; its “Webhooks” service allows developers to integrate other services on its platform with their IoT project(s) via simple web requests. In this work, the presented AI-embedded and Arduino MCU-based smart power meter prototype, which triggers a pre-specified IFTTT/Maker event when an identified real-time actionable insight is present, is designed and implemented in the described architecture with LINE Notify. Receiving push notifications from an LINE Notify official account is realized in the described architecture in Figure 1 and Figure 3. Providing a push notification service to IoT end users (clients), the PC-based data science analytics engine configured in Figure 3 can also suit Google-maintained Firebase Cloud Messaging (Google Cloud Messaging) to engage IoT clients across Android/iOS mobile devices. In the described architecture in Figure 3, data transmitted by or requested from the designed and implemented AI-embedded and Arduino MCU-based smart power meter prototype are over the HTTP [21]. To cloud storage/IoT data stores, a network-attached storage providing data access via the HTTP GET()/POST() methods for a heterogeneous group of IoT clients can be configured. Instead, an open IoT analytics platform, ThingSpeak^TM^, is conducted and suited in this work. ThingSpeak^TM^ is an open-source IoT platform with MATLAB^®^ analytics (by MathWorks^®^), which provides an API to be used by IoT clients to store IoT data to and retrieve IoT data from it over the HTTP [21].

As electrical appliances monitored in a residential environment in the described cloud analytics-assisted electrical EMS architecture in Figure 1, Figure 2 and Figure 3 are able to react to DR developed by utilities to shift electrical energy consumption patterns during peak load periods for DSM in an SG [3,4], the prototype developed in the architecture described above accommodates AI, the FCM clustering/piloting RBF-ANN, to identify electrical appliances monitored on-line without an intrusive deployment of smart plugs for monitored electrical appliances. The prototype that accommodates the FCM-piloting RBF-ANN to perform on-line load monitoring is presented below.

### 3.2. AI-Embedded and Arduino MCU-Based Smart Power Meters Prototype Designed and Implemented as Edge Analytics with Push Notification Service for DSM in an SG

**Figure 5:**
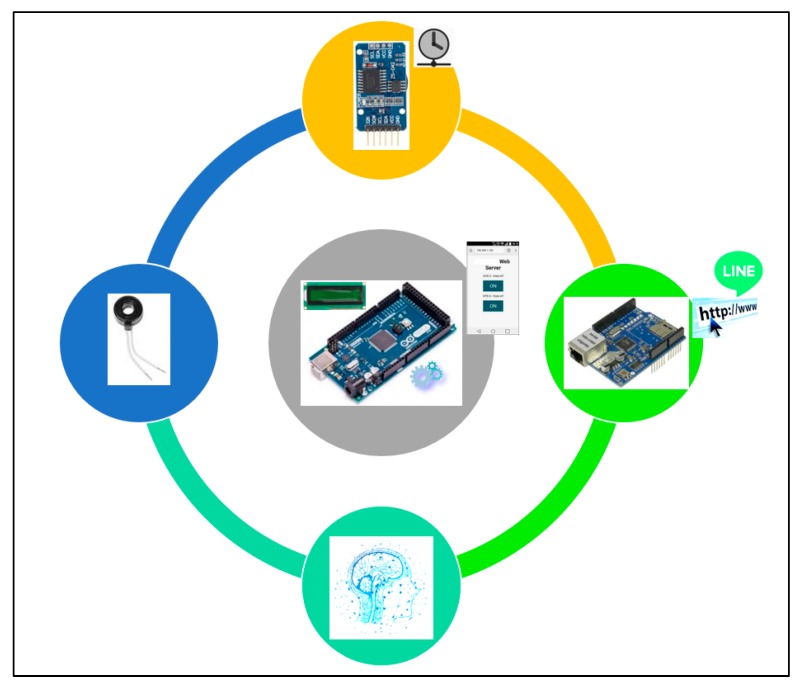
Presented smart AI across IoT (AIoT) edge analytics-empowered power meter prototype. The current sensor-based prototype is based on Arduino MCU providing a push notification service.

The AI-embedded and Arduino MCU-based smart power meter prototype designed and implemented as edge analytics in the described architecture with push notifications service is depicted in Figure 5. It can be seen in Figure 5 that the core/main entity of the prototype is based on the Arduino board. More specifically, Arduino MEGA 2560 [34,35] was chosen and used for the hardware/software design and implementation of the prototype in this work. Arduino MEGA 2560 is an open-source and inexpensive product, and it provides sufficient analog pins for its possible different future ideas in smart homes.

The general specification of Arduino MEGA 2560 is shown in Table 1. Arduino MEGA 2560 is an MCU board based on Atmel^®^ 8-bit ATmega2560 MCU, which is designed and conducted for more complex projects. It has 54 digital Input/Output (I/O) pins (of which 15 can be used for pulse width modulation (PWM)), 16 analog inputs, four Universal Asynchronous Receiver/Transmitter (UARTs) (hardware serial ports), a large memory space for coded Arduino sketch, a 16-MHz crystal oscillator, a Universal Serial Bus (USB) connection, a power jack, an ICSP (in-circuit serial programming) header, and a reset button. In an Arduino MCU, the flash memory, the program space, is where the coded Arduino sketch is stored. The SRAM (static random-access memory) is where the coded sketch creates and manipulates variables when it runs. The Electrically Erasable Programmable Read-Only Memory (EEPROM) is the memory space that programmers can use to store long-term data. The Arduino MEGA 2560 MCU is compatible with most shields designed for Arduino UNO and the former MCUs such as Arduino Duemilanove.

The Arduino MEGA 2560 MCU is programed in Arduino language. Arduino language is based on the C/C++ programming language, and it comes with a user-friendly IDE (integrated development environment) [36]. In this work, the designed and implemented prototype in Figure 5 is based on the Arduino MEGA 2560 MCU, which modules (1) a current transducer (CT) coil clipped on the live wire of electrical wiring and used to sense/measure currents for on-line load monitoring; (2) a WizNet W5100 hardwired TCP/IP embedded ethernet shield mounted and used to support internet connectivity; (3) a real-time clock (RTC) chip used to keep track of present time, as timestamps, via network time protocol (NTP) (all AI-embedded and Arduino MCU-based smart power meters distributed and deployed on-site for DSM in smart homes in an SG are time-synchronized); (4) a micro Secure Digital (SD) card used to store IoT data (an SD Library, “sdfatlib”, by William Greiman allows for reading data from and writing data to an SD card); and (5) AI, the commonly used BP-ANN model [8,16,17,18,19,21,23] and comparative FCM clustering/piloting RBF-ANN model, embedded/deployed on-site on the Arduino MEGA 2560 MCU and used as edge analytics to perform on-line load monitoring.

Wireless communication technology such as Bluetooth [37], ZigBee [38], and Wi-Fi [39,40,41] can be conducted and used for the presented prototype in the described architecture in this work. ZigBee (the Institute of Electrical and Electronics Engineers (IEEE) 802.15.4 standard) [38] is arguably the most popular technology for creating wireless sensor networks, and it was included in some of the latest commercial [40] and academic home automation cases, including water pump control in a smart fish farm with efficient energy consumption [15,42,43], and several others [39,40,44]. The new Android smartphone application that uses the open-source Massachusetts Institute of Technology (MIT) App Inventor 2 software to monitor voltage and current measurements in Reference [37] can also be conducted, developed, and used by the presented prototype in the described architecture. In this sense, the WizNet W5100 hardwired TCP/IP embedded ethernet shield mounted on the Arduino MEGA 2560 MCU for the design and implementation of the presented AI-embedded and Arduino MCU-based smart power meter prototype in this work can be replaced with a low-cost ESP8266 ESP-01 SoC (system on a chip) Wi-Fi microchip; an open-source firmware based on ESP8266 Wi-Fi-SoC NodeMCU in Reference [21] is used.

**Figure 6:**
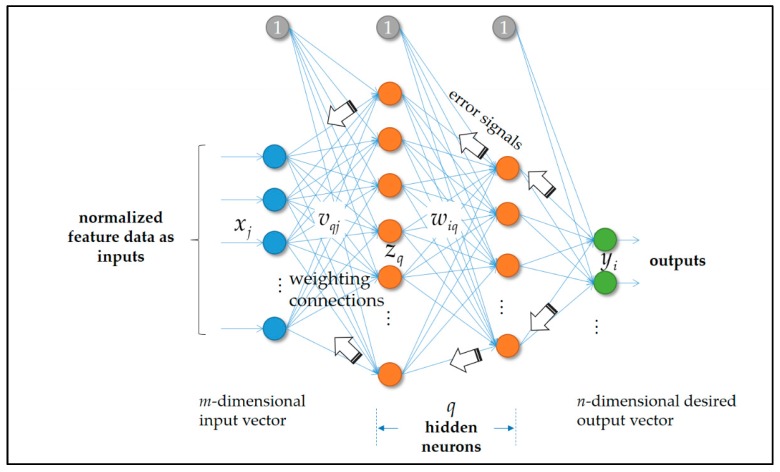
Structure of a feed-forward backpropagation (BP) artificial neural network (ANN) model used in this work, trained by a gradient-descent (GD) learning process in cloud analytics and compared, as a comparative study, with a fuzzy C-means (FCM) clustering/piloting radial basis function (RBF) ANN model.

**Figure 7:**
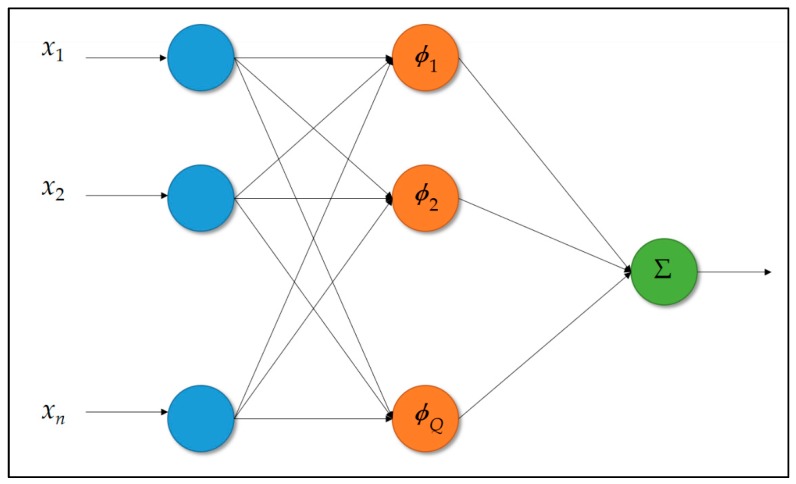
Structure of a feed-forward RBF-ANN used and compared with the BP-ANN model in this work.

In the presented AI-embedded and Arduino MCU-based smart power meter prototype in the described architecture in this work, the widely used BP-ANN model [8,16,17,18,19,21,23,45] in Figure 6 is constructed, trained in cloud analytics, and compared with an RBF-ANN model in Figure 7.

The RBF-ANN model in Figure 7 is integrated with an FCM clustering algorithm in this work. Both AI models in Figure 6 and Figure 7 are trained in cloud analytics and compared in this work; the superior AI model, well trained in cloud analytics, is then embedded/deployed on-site on the designed and implemented smart Arduino MCU-based power meter prototype as edge analytics. The AI-embedded and Arduino MCU-based smart power meter prototype presented in this section is used to perform on-line load monitoring as load identification for DSM in an SG.

The process of on-line load monitoring by the presented prototype for DSM in this work comprises feature extraction and load identification, as shown in Figure 2. In this work, real power (P in Watts) [20,21] and turn-on transient power [21] are extracted from monitored electrical appliances through feature extraction and are used as load signatures/electrical features to be trained in cloud analytics and identified by the presented smart Arduino MCU-based smart power meter prototype accommodating the superior AI model. The turn-on transient power is defined as real power consumption in which real power consumed by an electrical appliance plugged into the presented prototype and turned on is computed and captured. Electrical appliances will settle down. For feature extraction, current waveforms acquired by the CT from the time domain can be transformed, through fast Fourier transform (FFT) [46,47,48,49], into the frequency domain for current harmonics [20] as load signatures. FFT is an algorithm that performs discrete Fourier transform. In this work, the two different types of AI, the BP-ANN model [21,23] and FCM clustering/piloting RBF-ANN model, are conducted and compared. They are introduced below.

#### 3.2.1. Widely Used BP-ANN Model

In the biologically inspired and widely used BP-ANN model shown in Figure 6, artificial neurons with synaptic weights including biases (weighting connections) are arranged and fully connected in the input, hidden, and output layers. In this work, load signatures used as input data/training samples are fed through the network to be trained. During the training process of the BP-ANN network, the types of monitored electrical appliances indicated from actual output(s) of the network to be computed are compared with the desired/target value(s) in a supervisory manner, and the error(s) compared and computed are then fed back through the network to be trained. The BP-ANN network incrementally adjusting its weighting connections will ultimately be trained (the total error is systematically reduced, since the weighting connections are adjusted as the training process of the network proceeds). The adjustable parameters, the weighting connections of the BP-ANN network in Figure 6, include *v_qj_* and *w_iq_*. The weighting connections of the BP-ANN network constructed in this work with only one single hidden layer as an example in Figure 6 are updated according to Equations (1) and (2), which are updated during the backward-pass process of the whole training process of the network [8].

(1) $$\Delta w_{iq}=\eta [d_{i}-y_{i}][a^{\prime }(net_{i})][z_{q}]$$

In Equation (1), *η* is the learning rate, *d_i_* is the desired/target output of the *i*-th artificial neuron in the output layer of the BP-ANN network, *y_i_* is the computed output of the *i*-th artificial neuron in the output layer of the network, *a* denotes the user-specified activation function—an abstraction representing the rate of action potential firing in a biological neuron cell, which can be, for example, a continuous sigmoid-type function, and *net_i_* is computed and considered the net input of the *i*-th artificial neuron in the output layer of the BP-ANN network (from neurons outputted in the hidden layer during the forward-pass process of the whole training process of the network), which is usually a weighted sum of the inputs of the *i*-th artificial neuron. Furthermore, *z_q_*, $z_{q}=a\left(net_{q}\right)=a\left(\sum_{j=1}^{m}v_{qj}x_{j}\right)$, is the computed output of the *q*-th artificial neuron in the hidden layer of the network (its net input, a weighted-sum value, is computed when *x_j_* is presented during the forward-pass process).

(2) $$\Delta v_{qj}=\eta \sum_{i=1}^{n}\left[[d_{i}-y_{i}][a^{\prime }(net_{i})]w_{iq}\right]a^{\prime }(net_{q})x_{j}$$

The commonly used gradient-descent (GD) learning algorithm involving Equations (1) and (2) is conducted and used to train the BP-ANN network in this work. Equations (1) and (2) adopt the incremental approach in updating the weighting connections of the BP-ANN network; that is, the weighting connections of the network are changed after one training sample is presented and computed.

#### 3.2.2. FCM Clustering/Piloting RBF-ANN Model

The RBF-ANN model [50,51,52,53], a rather simple three-layer ANN model, conducted and used in this work, includes one input layer, one hidden layer involving radial basis functions to take on the role of non-linear activation functions, and one output layer, as shown in Figure 7. RBF-ANNs have several advantages in comparison to BP-ANN/multi-layer perceptron. For instance, they have conspicuous fast and high learning and generalization performance [8,50,53]. Also, there are few principal design factors to be determined for RBF-ANN compared with BP-ANN. As a result, the RBF-ANN model combined with FCM clustering [54,55,56] is conducted and used in this work. The FCM clustering, the fuzzy version of the known k-means clustering, is a partitioning-based clustering algorithm used in this work to design the RBF-ANN model in Figure 7. To design the RBF-ANN model in Figure 7, the first important step is to determine the parameters of each basis function in the hidden layer of the network. In this work, the radially symmetric Gaussian basis functions involving the center and spread parameters are used. The Gaussian basis functions used by the RBF-ANN model in this work are distributed, partially overlapped, and coarsely determined through the FCM clustering. Once the specification of the center and spread parameters of each Gaussian basis function is completed, a singular value decomposition (SVD) technique [50] is used to train the RBF-ANN model.

The two-stage approach used in this work to design the RBF-ANN model in Figure 7 is summarized below.

- Stage 1. FCM clustering is applied [54,55,56], with an on-site collected training dataset, to coarsely determine the center and spread parameters of Gaussian-type basis functions of the RBF-ANN model [50,51,52,53]. The Gaussian basis functions heuristically initialized are evenly spanned. The spread parameter of each of the Gaussian basis functions can be computed with *e*^−1^ from clustered data with their center mean.
- Stage 2. The SVD technique is used [50], with the on-site collected training dataset, to train the RBF-ANN model heuristically initialized in Stage 1. Once the FCM clustering/piloting RBF-ANN model is trained, in cloud analytics, with an acceptable level of performance, it is then deployed on-site on the presented smart Arduino MCU-based power meter prototype. Also, it is used to classify new data instances for on-line load monitoring in DSM.

The FCM clustering allows each datum to belong to two or more clusters, as demonstrated in fuzzy logic theory. The goal of the FCM clustering applied in this work is to find *c* cluster centers/codebook prototypes/centroids with respect to an on-site collected training dataset. The *c* centers found are used to roughly allocate Gaussian-type basis functions of the RBF-ANN model involving the center and spread parameters.

The FCM clustering algorithm used in this work for the RBF-ANN model initialized is given below.

**Step 1**.  For an on-site collected training dataset $ℑ={\left\{X_{k}{,}^{}d_{k}\right\}}_{k=1}^{Q}$, where there are *Q* input–output pairs of training samples, $X_{k}\in R^{n}$ and $d_{k}\in R$: fix *c* ∈ {2, 3, …, (*Q* − 1)}, set *m* ∈ (1, ∞), and initialize *U*^(0)^ ∈ *M_fc_*. Here, *M_fc_* = {*U* ∈ *V_cn_*|*u_ik_* ∈ interval [0, 1], 1 ≤ *i* ≤ *c*, 1 ≤ *k* ≤ *Q*; $\overset{c}{\underset{i=1}{\sum }}u_{ik}=1,\;\forall k\in \left\{1,\;2,\;\ldots ,\;Q\right\}$ is true}; *V_cn_* is the set of real *c* × *Q* matrices *U* = [*u_ik_*]; *u_ik_* is the membership value of $X_{k}$ that belongs to the *i*-cluster.
**Step 2**.  At iteration *l*, where *l* = 0, 1, 2, …, compute the *c* mean centers using Equation (3).
  (3) $$V_{i}=\frac{\sum_{k=1}^{Q}{\left(u_{ik}{}^{\left(l\right)}\right)}^{m}X_{k}}{\sum_{k=1}^{Q}{\left(u_{ik}{}^{\left(l\right)}\right)}^{m}},1\leq i\leq c$$
**Step 3**.  Update *U*^(*l*)^ to *U*^(*l* + 1)^ = [*u_ik_*^(*l* + 1)^] using Equation (4).
  (4) $$u_{ik}{}^{\left(l\right)}=\frac{1}{\sum_{j=1}^{c}{\left(\frac{\left||X_{k}-V_{i}{}^{\left(l\right)}|\right|}{\left||X_{k}-V_{j}{}^{\left(l\right)}|\right|}\right)}^{\frac{2}{m-1}}},1\leq i\leq c,1\leq k\leq Q$$
**Step 4**.  If ||*U*^(*l* + 1)^ − *U*^(*l*)^|| is less than or equal to a pre-specified tolerance, stop; otherwise, set *l* = *l* + 1 and go to **Step 2**.

The cluster centers identified, by Equation (3), through the FCM clustering are used as the center parameters of the Gaussian basis functions of the RBF-ANN model trained by the SVD technique as described below.

According to the same training dataset $ℑ={\left\{X_{k},\;d_{k}\right\}}_{k=1}^{Q}$, the goal of the training process of the RBF-ANN model is to search for map *f* that takes each input $X_{k}$ (k=1, …, Q) and then maps it exactly onto its desired/target output $d_{k}$: $f(X_{k})=d_{k}$. With the purpose of mapping $f(X_{k})=d_{k}$ to be learned through the training process of the RBF-ANN model, the RBF-ANN model assumes a set of exact *q* non-linear Gaussian-type basis functions $\phi (\|X_{k}-\mu_{i}\|)$ whose argument involves a Euclidean distance metric ${\|X}_{k}-\mu_{i}\|$. The Euclidean distance metric measures the distance between the *k*-th inputted input $X_{k}$ and the *i*-th center (cluster mean) $\mu_{i}$, where *i* = 1, 2, …, *q* (= *c*) and $\mu_{i}\in R^{n}$. In this work, *q* is equal to *c*; the *c* cluster means in Equation (3) are found by the FCM clustering that was given previously. The map *f* above is then generated with a weighted linear superposition of the *q* non-linear basis functions, as shown in Equation (5).

(5) $$f(X)=\sum_{i=1}^{q}w_{i}\phi ({\|X}_{k}-\mu_{i}\|)$$

In Equation (5), *w_i_* is the *i*-th weight coefficient of the RBF-ANN model that needs to be trained.

The map *f* in Equation (5) is solved in a least squares sense. Toward this end, the familiar squared error function that computes the squared error that is summed over all *Q* training samples is introduced in Equation (6).

(6) $$\varepsilon =\frac{1}{2}\sum_{k=1}^{Q}[d_{k}-\sum_{i=1}^{q}w_{i}\phi ({\|X}_{k}-\mu_{i}\|){]}^{2}$$

To obtain the optimal weight coefficients in a least squares sense, we differentiate Equation (6) with respect to $w_{i}$, and set it equal to zero as shown in Equation (7).

(7) $$\sum_{k=1}^{Q}\phi_{ki}(\sum_{l=1}^{q}w_{l}\phi_{kl})=\sum_{k=1}^{Q}d_{k}\phi_{ki}$$

By the following matrix definitions:

$\Phi =\left[\begin{array}{ccc} \varphi_{11} & \ldots & \varphi_{1q}\\ \varphi_{21} & \ldots & \varphi_{2q}\\ \vdots & \ddots & \vdots \\ \varphi_{Q1} & \ldots & \varphi_{Qq} \end{array}\right]$, *D* = ${\left[d_{1}{,}^{}\ldots {,}^{}d_{Q}\right]}^{T}$, and *W* = ${\left[w_{1}{,}^{}\ldots {,}^{}w_{q}\right]}^{T}$, Equation (7) is recast into a matrix form of Equation (8).

(8) $$\left(\Phi^{T}\Phi \right)W=\Phi^{T}D$$

Finally, the weight vector, *W*, in Equation (8) is solved by the SVD technique [50,57,58], and it is given in Equation (9).

(9) $$W={\left(\Phi^{T}\Phi \right)}^{-1}\Phi^{T}D=\Phi^{*}D$$

In Equation (9), $\Phi^{*}$, a *q*-by-*Q* matrix, is the pseudo-inverse [50].

In this work, *q* (= *c*) non-linear Gaussian basis functions are $\varphi \left(\left\|X_{k}-\mu_{i}\right\|\right)=\exp\left(-\frac{\|X_{k}-\mu_{i}\|{}^{2}}{2\sigma^{2}}\right)$. Assuming that the Gaussian basis functions are centered, by the *c* cluster means in Equation (3), at ${\left\{\mu_{i}\right\}}_{i=1}^{q}$, we define the maximum distance *α* between any of the chosen center parameters as $\alpha =\underset{1\leq i,j\leq q}{max}\left(\left\|\mu_{i}-\mu_{j}\right\|\right)$. Then, the spread parameters, σ, of the Gaussian basis functions are heuristically initialized by Equation (10) [50].

(10) $$\sigma =\frac{\alpha }{\sqrt{2q}}$$

Equation (5) admits the RBF-ANN model hybridized with the FCM clustering, as shown in Figure 7. The FCM clustering is used to find *c* cluster centers from an on-site collected training dataset, which are used as the center parameters of the Gaussian basis functions of Equation (5), and each spread parameter of the Gaussian basis functions is computed according to Equation (10). Algorithm 1 gives the pseudo code of code implementation of the FCM clustering/piloting RBF-ANN model, in this work, trained in cloud analytics and embedded/deployed on-site on the presented smart Arduino MCU-based power meter prototype for on-line load monitoring in DSM.

## 4. Proof-of-Concept Demonstration

### 4.1. Demo Prototype and Evaluation

**Figure 8:**
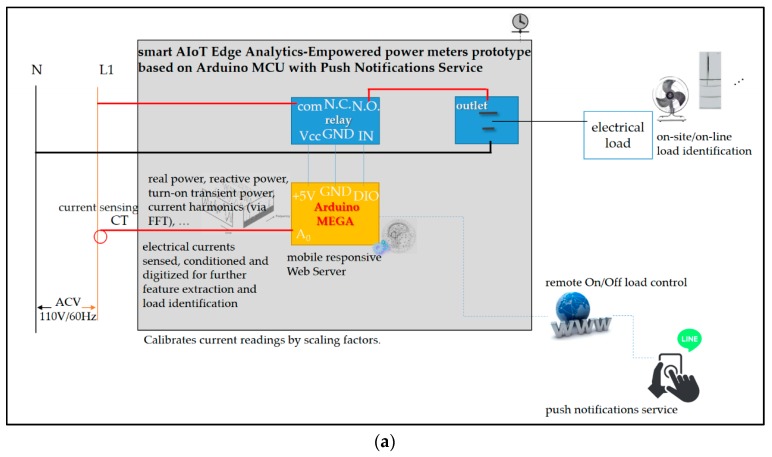

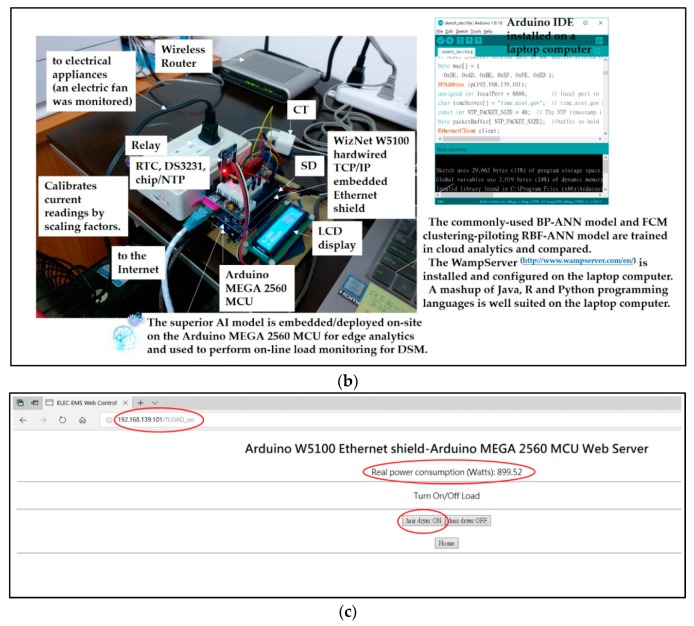
Proof-of-concept demonstration of the described cloud analytics-assisted electrical EMS architecture with the presented AI-embedded and Arduino MCU-based smart power meter prototype, the smart AIoT edge analytics-empowered power meters prototype based on Arduino MCU, as edge analytics for on-line load monitoring as load identification in DSM in this work. (**a**) Sketch of the designed and implemented prototype installed in a practical environment for an electrical network topology and used as a smart electrical outlet/wall socket. (**b**) Experimental set-up of the presented prototype in the described architecture in the realistic laboratory environment. (**c**) Electrical EMS web control: the mobile responsive web server configured on the Arduino MEGA 2560 MCU mounted with the Arduino W5100 ethernet shield and wired with a relay for remote on/off load control.

In this section, the cloud analytics-assisted electrical EMS architecture empowered by the AI-embedded and Arduino MCU-based smart power meter prototype as edge analytics for on-line load monitoring in DSM in this work is demonstrated and validated experimentally. Figure 8 shows the proof-of-concept demonstration of the described cloud analytics-assisted electrical EMS architecture with the presented AI-embedded and Arduino MCU-based smart power meter prototype. The presented prototype is designed and implemented as edge analytics for on-line load monitoring in the described architecture in this work, which is installed in a realistic laboratory environment for an electrical network topology and used as a smart electrical outlet/wall socket as shown in Figure 8a.

AI considered in this work includes the BP-ANN model and the comparative FCM clustering/piloting RBF-ANN model. The two AI models are trained in cloud analytics on a laptop computer suited, configured, and acted as the data science analytics engine in Figure 3. The laptop computer suited, configured, and acted as cloud analytics is shown in Figure 8b. In Figure 8a,b, the AI-embedded and Arduino MCU-based smart power meter prototype depicted in Figure 5 are shown. We assembled hardware and software of the smart Arduino MEGA 2560 MCU-based power meter prototype with its mounted Arduino W5100 Ethernet shield, in order to (1) acquire electrical currents measured by the CT; (2) identify load signature/electrical features, based on embedded AI, extracted from electrical appliances monitored for on-line load monitoring/load identification in DSM in this work; (3) realize remote on/off load control based on a mobile responsive web server configured on the Arduino MEGA 2560 MCU mounted with the Arduino W5100 ethernet shield and wired with a relay; and (4) provide a mobile push notification service to end users for user-centric IoT applications in DSM. The mobile responsive web server configured on the Arduino MEGA 2560 MCU and used to realize electrical EMS web control is shown in Figure 8c. The push notification service provided is shown later. In this experimentation, the two AI models, the BP-ANN model and the FCM clustering/piloting RBF-ANN model, are trained, in R language installed on the laptop computer, in cloud analytics. Then, the superior AI model is embedded/deployed on-site on the Arduino MEGA 2560 MCU as edge analytics for on-line load monitoring/load identification, auto-labeling of monitored electrical appliances [21,23], in DSM in this work (refer to Figure 2 and Figure 8a). On-line load identification in this work can be developed on a daily basis of appliance-level load identification, as appliance-level load identification is disaggregated from whole-house load data with no intrusive deployment of smart plugs installed for electrical appliances monitored. The BP-ANN model is trained according to Equations (1) and (2). The FCM clustering/piloting RBF-ANN model, an AI model compared with the BP-ANN model, is trained according to Algorithm 1. Both AI models trained, compared, and used to identify electrical appliances in this experimentation are shown later. How to size the burden resistor used by the AI-embedded and Arduino MCU-based smart power meter prototype in Figure 8b to convert CT current into a voltage reference can be found in Reference [59].

**Algorithm 1.** Algorithm for the FCM clustering/piloting RBF-ANN model.Applying the FCM clustering on an on-site collected training dataset $ℑ={\left\{X_{k}{,}^{}d_{k}\right\}}_{k=1}^{Q}$ to coarsely determine the center and spread parameters of the *q* (= *c* in the FCM) Gaussian basis functions of the RBF-ANN model of Equation (5). ↬
**Specify** values: the number of clusters *c*, the degree of fuzziness *m* > 1, and a tolerance to be set. ↬
**Generate** the *c* cluster centers randomly. *l* ← 0 ↬
**Repeat** **Compute** the *c* cluster centers, using Equation (3). **Update** the membership matrix, *U*, using Equation (4). *l* ← *l* + 1, **until** the tolerance, ||*U*^(*l*+1)^ − *U*^(*l*)^||, is approximately met. ↬
**Return** the resulting *c* cluster centers found with the membership matrix *U.* ↬
**Center** the Gaussian basis functions of Equation (5) at the resulting *c* cluster centers found through the FCM clustering. ↬
**Compute** the spread parameters of the Gaussian basis functions of Equation (5), using Equation (10). ^1^Use the SVD technique, with the on-site collected training dataset, to train the RBF-ANN model of Equation (5). Its Gaussian basis functions are heuristically initialized by the FCM clustering. ↬
**Train** Equation (5), by Equation (9), in cloud analytics, to get *W*. ↬
**Deploy** the well-trained AI model on-site on the presented smart Arduino MCU-based power meter prototype for on-line load monitoring in DSM. (^1^ The spread parameter of each of the Gaussian basis functions can be computed with *e*^−1^ from clustered data with their cluster mean/the membership matrix.)

In Figure 8c, network address translation (NAT), port forwarding/port mapping, is configured and used to allow remote computers/mobile devices to connect to the privately configured and local area network (LAN)-dominated mobile responsive web server via the internet, where the external interface of the NAT is configured with a public IP (Internet Protocol) address. In computer networking, NAT redirects HTTP requests based on a translation of a private IP address to a public IP address.

To feature extraction in this experimentation, (instantaneous) currents are acquired, and electrical features, real power (RMSPower) and turn-on transient power (peakPower), are extracted. RMSPower and peakPower are used as the input variables of the BP-ANN and FCM clustering/piloting RBF-ANN models; the electrical features are fed into the two AI models and learned. Current harmonics by FFT can be calculated and used as additional electrical features. That is, the electrical features learned can be P/RMSPower, peakPower, and/or current harmonics. To calculate current harmonics by FFT, the sampling frequency used by Arduino MCU has to be customized/modified according to the Nyquist–Shannon sampling theorem where the sampling frequency to frequently sample a signal you are trying to acquire needs to be at least twice the frequency of the signal you are trying to acquire. For load identification demonstrated in this experimentation, the BP-ANN and FCM clustering/piloting RBF-ANN models are used to learn and identify RMSPower and peakPower extracted from electrical appliances monitored for on-line load monitoring in DSM in this work.

In the proof-of-concept demonstration shown in this work, the ThingSpeak^TM^ IoT platform [21,23,60] that provides free cloud storage for IoT data stores is used. Table 2 shows the ThingSpeak^TM^ update executed by the presented prototype and used to upload electrical features, RMSPower and peakPower, defined above and summarized in Table 3, to the ThingSpeak^TM^ platform for data stores.

Table 3 shows the field of RMSPower and peakPower gathered from the presented prototype and uploaded through the internet to ThingSpeak^TM^.

**Figure 9:**
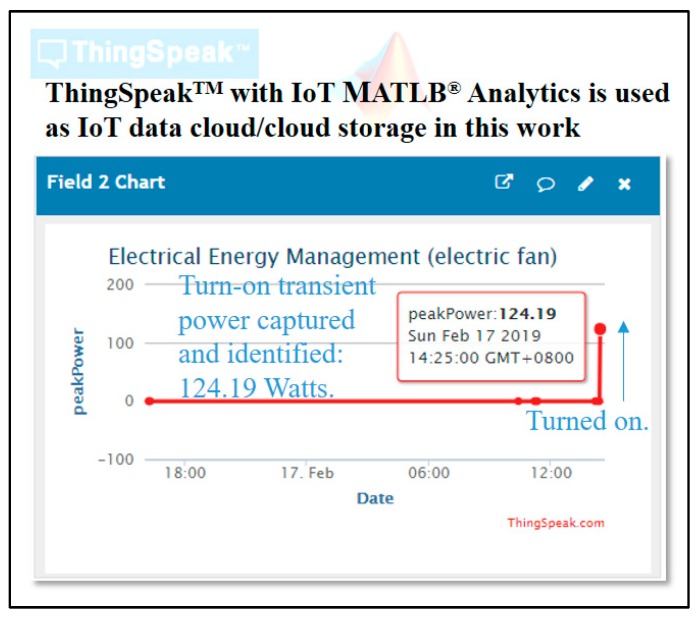
Data visualization by the open ThingSpeak^TM^ IoT platform for electrical energy management in this experimentation. The turn-on transient power, peakPower, consumed by an electric fan and identified by the presented AI-embedded and Arduino MCU-based smart power meter prototype, was 124.19 W.

The open ThingSpeak^TM^ IoT platform also provides a simple web page/dashboard to IoT clients for data visualization. As shown in Figure 9, data identified by the presented AI-embedded and Arduino MCU-based smart power meter prototype are visualized in ThingSpeak^TM^.

In this experimentation, the BP-ANN model in References [21,23] and the FCM clustering/piloting RBF-ANN model in this work are compared. Electrical appliances monitored and identified by the two AI models in this experimentation include an electric fan (~115 W) and a hair dryer (~900 W); the electrical features extracted from the monitored electrical appliances and used to train the two different types of AI models are RMSPower and peakPower. A network structure of 2–3–3 was structured for the BP-ANN model in Reference [23], and it was trained, by the GD algorithm, in cloud analytics. A network structure of 2–8–3 was used for the BP-ANN model in Reference [21], and it was also trained, by the GD algorithm, in cloud analytics. In the FCM clustering/piloting RBF-ANN model trained according to Algorithm 1, for the FCM clustering used to heuristically initialize the RBF-ANN model, the number of clusters, *c*, was pre-specified as 5, the degree of fuzzification, *m*, was set to 2, the maximum number of iterations executed was 100, and the Euclidean metric in Equation (4) was considered. The cluster centers found by the FCM clustering and used to allocate the Gaussian basis functions of Equation (5) in the RBF-ANN model are shown in Table 4. The spread parameters of the Gaussian basis functions, which are computed by Equation (10), were 570.28. The RBF-ANN model heuristically initialized by the FCM clustering was fine-tuned, by Equation (9), in cloud analytics.

In this experimentation, the superior AI model, the well-trained FCM clustering/piloting RBF-ANN model compared with the two BP-ANN models, is shown in Table 5. The superior FCM clustering/piloting RBF-ANN model was then embedded/deployed on-site on the presented smart Arduino MEGA 2560 MCU-based power meter prototype as edge analytics for on-line load monitoring in DSM in this work.

In this experimentation, the Arduino sketch coded with the superior FCM clustering/piloting RBF-ANN model used 33,750 bytes (13%) of the maximum program storage space of 253,952 bytes; the global variables used 2524 bytes (30%) of the maximum dynamic memory of its maximum 8192 bytes, leaving 5668 bytes for local variables. On-site measured data were identified by the superior FCM clustering/piloting RBF-ANN model for on-line load monitoring in DSM in this work, which were written to an SD card and uploaded to the ThingSpeak^TM^ platform for IoT data stores and data visualization. The feature space was of RMSPower and peakPower extracted from the electrical appliances monitored and identified by the superior FCM clustering/piloting RBF-ANN model.

**Figure 10:**
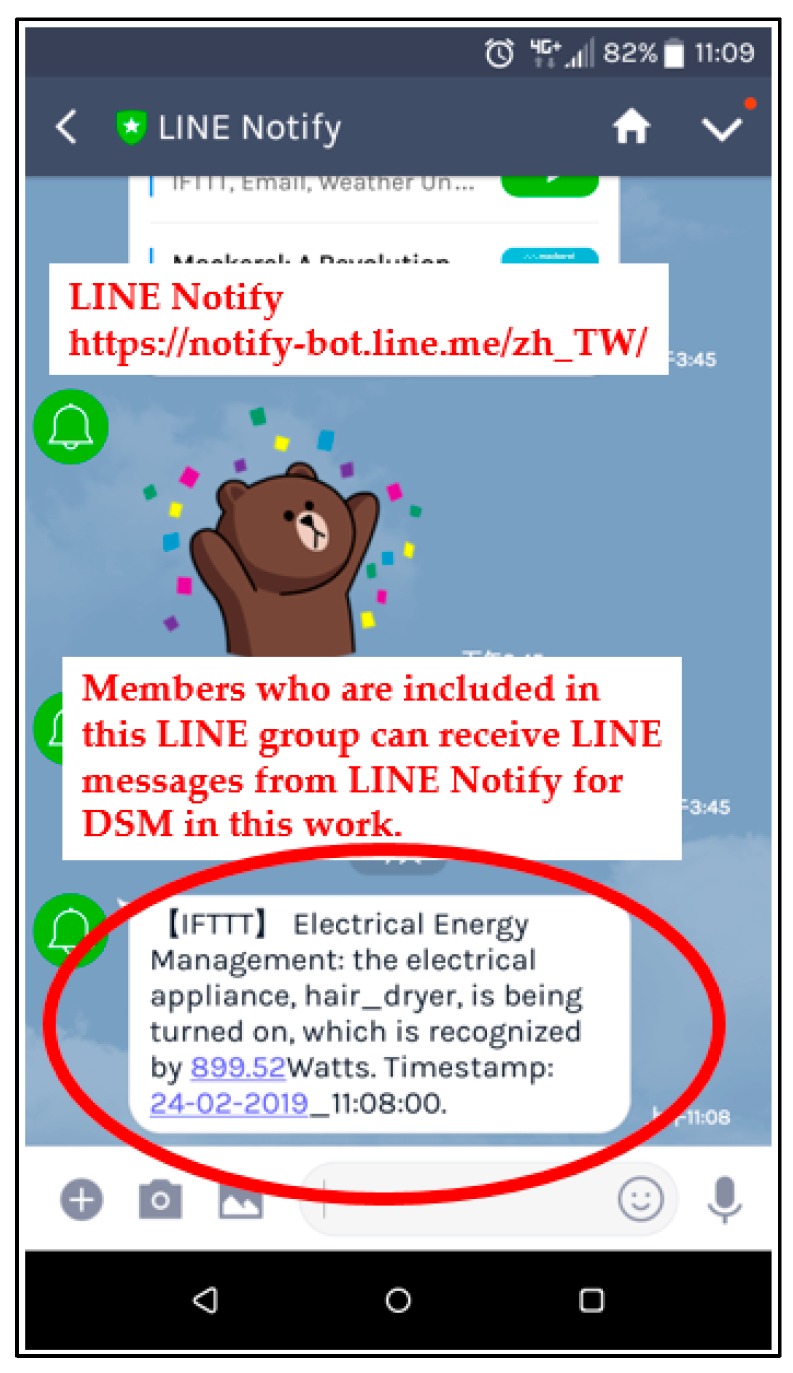
Received LINE Notify (https://notify-bot.line.me/zh_TW/) message based on the IFTTT (if this, then that) push notification service. The appliance event was pre-specified, identified, and then triggered for the monitored hair dryer.

Table 6 summarizes the overall load identification rates obtained, as a comparative study, in this work. The overall load identification rate of 99.12% was achieved by the FCM clustering/piloting RBF-ANN model, for load identification in this experimentation. For the push notification service in Figure 1, Figure 3, and Figure 8a, Figure 10 shows the received LINE Notify message based on the IFTTT push notification service. As shown in Figure 10, for the push notification service via IFTTT setting up its own/specified applet, the LINE message was received when an appliance event pre-specified in IFTTT, identified by the presented AI-embedded and Arduino MCU-based smart power meter prototype, and then triggered through Webhooks, was published by Webhooks. IFTTT (https://ifttt.com/line & https://notify-bot.line.me/zh_TW/) conducted in this work is an easy and free way to get your apps and devices working together, which provides free web-based services to create chains and applets of simple conditional statements. LINE (https://line.me/zh-hant/ and https://linecorp.com/zh-hant/) used in this work is a global messaging app used in over 230 countries and regions, which offers fun and free voice, video, and chat communication across multiple platforms. A webhook in IFTTT is a method of augmenting or altering the behavior of a web page/web application with custom callbacks.

### 4.2. Discussion

Figure 8 shows the proof-of-concept demonstration of the described cloud analytics-assisted electrical EMS architecture with the presented AI-embedded and Arduino MCU-based smart power meter prototype as edge analytics for on-line load monitoring and load identification in DSM in this work. Table 6 shows the overall load identification rate of the two different types of AI models accommodated and compared in the demonstrated architecture. As shown in Table 6, the FCM clustering/piloting RBF-ANN model presented in this work outperformed the two BP-ANN models applied in References [21,23]. The RBF-ANN model, integrated with the FCM clustering and compared with the two BP-ANN models, demonstrated its conspicuous fast and high learning and generalization performance, and there were few principal design factors that needed to be considered and determined for the RBF-ANN model. The two BP-ANN models in References [21,23] involved the forward and backward propagation processes in the GD algorithm and Equations (1) and (2), whereas the RBF-ANN model admitted by Equation (5) involved Equation (9) for its training process. The performance of the two BP-ANN models that were applied in References [21,23], compared to the RBF-ANN model, depended on three principal design factors [8,50,53]: (1) the architecture of the neural network structured, (2) the types of transfer functions specified, and (3) the training algorithm of learning from data used to train weighting connections of the structured neural network, which need to be considered and determined through trial and error. As pointed out in References [8,50,53] and shown in Table 6, in contrast to BP-ANN, RBF-ANN owns several advantages. For the partitioning clustering process conducted for the RBF-ANN model in this work, data collected on-site in a field of interest can be clustered through the nearest-neighbor clustering mechanism [7] in an unsupervised/self-organizing manner, where there will be no need to pre-specify the number of cluster centers, *c*, to be discovered ahead of time. In summary, the cloud analytics-assisted electrical EMS architecture empowered by the AI-embedded and Arduino MCU-based smart power meter prototype was demonstrated in this section. Moreover, the AI-embedded and Arduino MCU-based smart power meter prototype presented, as edge analytics, in the demonstrated architecture worked with a third-party push notification service, as shown in Figure 10. In the demonstrated architecture, for feature extraction processed through time-domain analysis, electrical features, RMSPower and peakPower, were extracted from electrical appliances monitored by the presented prototype. For load identification addressed by the two data-driven AI models, BP-ANN and FCM clustering/piloting RBF-ANN, the superior FCM clustering/piloting RBF-ANN model, advanced AI, compared with the BP-ANN model, was deployed on-site on the presented prototype and used to perform on-line load monitoring as load identification/auto-labeling of electrical appliances in DSM in this work. The different AI methods were trained, with a comparative analysis, in cloud analytics, and the superior AI was then embedded/deployed on-site on an IoT end device as edge analytics. This is necessary for a next-generation AMI/smart sensing infrastructure, as IoT end devices such as power meters deployed in fields of interest (for DSM) in an SG should be aware of interpretable and real-time actionable data insights at IoT sources. In the future, prognostics and health management (PHM) in fog-cloud analytics will be developed for Industry 4.0 applications, where data-driven PHM aims to predict the time, remaining useful life, at which a system or components of that system will no longer perform its or their intended function. It will be based on feature analysis in which electrical waveforms acquired from the time domain are transformed, through FFT, into the frequency domain with their past trends. In this sense, the presented prototype in the described architecture in this work can be further developed for and accommodated with preventative maintenance in DSM. For cloud analytics in the described architecture, a scalable cloud analytics engine spanning multiple technologies including graphics processing units (GPUs), message passing interface (MPI), and parallel NetCDF will be developed for more high-performance cloud analytics.

## 5. Conclusions and Future Work

Electrical energy forms an indispensable part of today’s modern society; people’s lives would be impossible without the aid of electricity. To meet continuously increasing electrical energy demands requested from downstream sectors of an SG, EMSs monitor and manage industrial, commercial, and residential electrical appliances efficiently in response to DR signals in DSM. In this work, a cloud analytics-assisted electrical EMS architecture empowered by an AI-embedded and Arduino MCU-based smart power meter prototype as edge analytics was described. Moreover, the AI-embedded and Arduino MCU-based smart power meter prototype presented, as edge analytics, in the described architecture worked with a third-party push notification service. In the described architecture, for feature extraction processed through time-domain analysis, electrical features, real power and turn-on transient power consumption, were extracted from electrical appliances monitored by the presented prototype; for load identification addressed by data-driven AI models, BP-ANN and FCM clustering/piloting RBF-ANN, the superior FCM clustering/piloting RBF-ANN model, compared with the BP-ANN model, was deployed on-site on the presented prototype and used to perform on-line load monitoring in DSM in this work. The BP-ANN model and the FCM clustering/piloting RBF-ANN model were used, as a comparative proof-of-concept demonstration, in this work. The activation of the RBF-ANN model compared to the BP-ANN model and hybridized with FCM clustering was not sigmoid, but radially symmetric. Therefore, information was represented locally in the neural network.

The practicality of the described architecture with the presented prototype in this work was based on a proof-of-concept demonstration. As the experimentation reported in this work shows, the described cloud analytics-assisted electrical EMS architecture having the presented AI-embedded and Arduino MCU-based smart power meter prototype as edge analytics for on-line load monitoring in DSM is feasible and workable. Different AI methods were trained, with a comparative analysis, in cloud analytics, and the superior AI was then embedded/deployed on-site on an IoT end device as edge analytics. This is necessary for a next-generation AMI/smart sensing infrastructure, as IoT end devices such as power meters deployed in fields of interest in an SG should be aware of interpretable and real-time actionable data insights at IoT sources.

In the future, a scalable cloud analytics engine spanning multiple technologies including GPUs, MPI, and parallel NetCDF will be developed for more high-performance cloud analytics. PHM in fog-cloud analytics will also be developed for Industry 4.0 applications, where data-driven PHM aims to predict the time, remaining useful life, at which a system or components of that system will no longer perform its or their intended function. It will be based on feature analysis in which electrical waveforms acquired from the time domain are transformed, through FFT, into the frequency domain with their past trends. In this sense, the presented prototype in the described architecture in this work can be further developed for and accommodated with preventative maintenance in DSM.

## Figures and Tables

**Table 1:** Technical specification of Arduino MEGA 2560 [34,35].

Microcontroller	ATmega 2560
Operating voltage	5 V
Input voltage (recommended)	7–12 V
Input voltage (limit)	6–20 V
Digital Input/Output (I/O) pins	54 (of which 15 provide Pulse Width Modulation (PWM) output)
Analog input pins	16
Direct Current (DC) current per I/O pin	20 mA
DC current for 3.3-V pin	50 mA
Flash memory	256 kB (8 kB used by its bootloader)
Static Random Access Memory (SRAM)	8 kB
Electrically Erasable Programmable Read-Only Memory (EEPROM)	4 kB
Clock speed	16 MHz
LED_BUILTIN, the number of the pin to which the on-board LED is connected	13
Length × width	101.52 mm × 53.3 mm

**Table 2:** ThingSpeak^TM^ update executed by the presented prototype.

/* … */
byte server[[] = {184, 106, 153, 149}; // IP Address (or api.thingspeak.com) for the ThingSpeak (https://thingspeak.com/)
String writeAPIKey = “E18Q*X*TY**8A*4U”; // Write API Key for a ThingSpeak Channel
…
…
updateThingSpeak(“field1=” + String(RMSPower) + “&field2=” + String(peakPower));
…
…
void updateThingSpeak(String tsData) { // RMSPower, peakPower, …, more ^1^

if (client.connect(server, 80)) {
Serial.println(F(“Connected to ThingSpeak...”));
client.print(“POST /update HTTP/1.1\n”);
client.print(“Host: api.thingspeak.com\n”);
client.print(“Connection: close\n”);
client.print(“X-THINGSPEAKAPIKEY: “+writeAPIKey+”\n”);
client.print(“Content-Type: application/x-www-form-urlencoded\n”);
client.print(“Content-Length: ”);
client.print(tsData.length());
client.print(“\n\n”);
client.print(tsData);
}
…

}
…

^1^ The current readings are calibrated by scaling factors [59] for power computation.

**Table 3:** Field of RMSPower and peakPower (turn-on transient power consumption) in ThingSpeak^TM^.

Channel	Field	Physical Meaning/Sensor
Electrical energy management	Field1 (RMSPower)	Real power/CT
	Field2 (peakPower)	Turn-on transient power/CT

**Table 4:** Cluster means found through the fuzzy C-means (FCM) clustering in this experimentation and used as center parameters of the Gaussian basis functions of the radial basis function (RBF) artificial neural network (ANN) model to be heuristically initialized.

RMSPower	peakPower
0	0
901.30	901.30
0	901.30
112.58	123.22
97.45	123.31

**Table 5:** Well-trained FCM clustering/piloting RBF-ANN model embedded/deployed on-site on the Arduino MEGA 2560 MCU as edge analytics for on-line load monitoring in DSM in this work.

*W*			
	0	0.5311	−14.9229
	0	1.1154	−0.5784
	0	−0.2486	−1.8118
	0	−0.9397	−4.3011
	0	0.3614	20.3958

**Table 6:** Load identification rates obtained, as a comparative study, in this work.

	The RBF-ANN Model Hybridized with the FCM Clustering in This Work ^1^	The BP-ANN Model in Reference [21] ^2^	The BP-ANN Model in Reference [23] ^3^
Overall Load Identification Rate (%) ^4^	99.12	99.12	94.12

^1^ The elapsed time consumed through the whole process of the FCM clustering/piloting RBF-ANN model was 0.255879 s, where (1) the number of cluster centers, *c*, found by the FCM clustering and used to heuristically initialize the 2–5–3 network structure of the RBF-ANN model was 5; 2) λ, a regularization parameter used to control the trade-off between the closeness to data fitted and the smoothness of the Tikhonov regularization called a stabilizer that forces the approximation to become as smooth as possible [50], was 0.05; and (3) only one data instance was misidentified. The output, the load indicator, of all the three ANN models compared in this work, was based on the winner-takes-all principle considering a threshold of 0.5 pre-specified. The output of all three ANN models was, in size, extensible with {1, 2, 3, ..., *n* + 1} for more electrical appliances monitored and identified by the presented prototype in the described architecture, where *n*, a value of 2 in this experimentation, is the total number of monitored electrical appliances. ^2^ A 2–8–3 network structure of the BP-ANN model was configured. ^3^ A 2–3–3 network structure of the BP-ANN model was configured. ^4^ A total of 102 data were measured are identified in this experimentation.

## References

[1] Hussain H.M., Javaid N., Iqbal S., Hasan Q.U., Aurangzeb K., Alhussein M. (2018). An efficient demand side management system with a new optimized home energy management controller in smart grid. Energies.

[2] Fadlullah Z.M., Quan D.M., Kato N., Stojmenovic I. (2014). GTES: An optimized game-theoretic demand-side management scheme for smart grid. IEEE Syst. J..

[3] Lin Y.H., Tsai M.S. (2015). An advanced home energy management system facilitated by nonintrusive load monitoring with automated multiobjective power scheduling. IEEE Trans. Smart Grid.

[4] Lin Y.H., Hu Y.C. (2018). Residential consumer-centric demand-side management based on energy disaggregation-piloting constrained swarm intelligence: Towards edge computing. Sensors.

[5] Veras J.M., Silva I.R.S., Pinheiro P.R., Rabêlo R.A.L. (2018). Towards the handling demand response optimization model for home appliances. Sustainability.

[6] Veras J.M., Silva I.R.S., Pinheiro P.R., Rabêlo R.A.L., Veloso A.F.S., Borges F.A.S., Rodrigues J.J.P.C. (2018). A multi-objective demand response optimization model for scheduling loads in a home energy management system. Sensors.

[7] Lin Y.H. (2018). Design and implementation of an IoT-oriented energy management system based on non-intrusive and self-organizing neuro-fuzzy classification as an electrical energy audit in smart homes. Appl. Sci..

[8] Lin Y.H., Hu Y.C. (2018). Electrical energy management based on a hybrid artificial neural network-particle swarm optimization-integrated two-stage non-intrusive load monitoring process in smart homes. Processes.

[9] Li W.X., Logenthiran T., Phan V.T., Woo W.L. (2018). Implemented IoT based self-learning home management system (SHMS) for Singapore. IEEE Internet Things J..

[10] Taoa M., Zuo J., Liu Z., Castiglione A., Palmieri F. (2018). Multi-layer cloud architectural model and ontology-based security service frame-work for IoT-based smart homes. Future Gener. Comput. Syst..

[11] Froiz-Míguez I., Fernández-Caramés T.M., Fraga-Lamas P., Castedo L. (2018). Design, implementation and practical evaluation of an IoT home automation system for fog computing applications based on MQTT and ZigBee-WiFi sensor nodes. Sensors.

[12] Chen Y.D., Zulfan Azhari M., Leu J.S. Design and implementation of a power consumption management system for smart some over fog-cloud computing. Proceedings of the 2018 3rd International Conference on Intelligent Green Building and Smart Grid (IGBSG).

[13] Moghaddam M.H.Y., Alberto L.G. (2018). A fog-based internet of energy architecture for transactive energy management systems. IEEE Internet Things J..

[14] Bonomi F., Milito R., Zhu J., Addepalli S. Fog computing and its role in the Internet of Things. Proceedings of the 1st edition of the MCC workshop on Mobile cloud computing (MCC’12).

[15] Shojafar M., Cordeschi N., Baccarelli E., Hassan Q.F. (2016). Resource scheduling for energy-aware reconfigurable Internet data centers. Innovative Research and Applications in Next-Generation High Performance Computing.

[16] Yang H.T., Chang H.H., Lin C.L. Design a neural network for features selection in non-intrusive monitoring of industrial electrical loads. Proceedings of the 11th International Conference on Computer Supported Cooperative Work in Design.

[17] Chang H.H., Lin C.L., Weng L.S. Application of artificial intelligence and non-intrusive energy-managing system to economic dispatch strategy for cogeneration system and utility. Proceedings of the 13th International Conference on Computer Supported Cooperative Work in Design.

[18] Chang H.H. (2012). Non-intrusive demand monitoring and load identification for energy management systems based on transient feature analyses. Energies.

[19] Chang H.H., Chen K.L., Tsai Y.P., Lee W.J. (2012). A new measurement method for power signatures of nonintrusive demand monitoring and load identification. IEEE Trans. Ind. Appl..

[20] Agyeman K.A., Han S., Han S. (2015). Real-time recognition non-intrusive electrical appliance monitoring algorithm for a residential building energy management system. Energies.

[21] Lin Y.H. (2019). Novel smart home system architecture facilitated with distributed and embedded flexible edge analytics in demand-side management. Int. Trans. Electr. Energy Syst..

[22] Lin Y.H., Lin W.C., Cheng Y.C., Yeh C.J., Chen Y.C., Li T.Y. Developing a cloud intelligence-based energy management architecture facilitated with embedded edge analytics for energy conservation in demand-side management. Proceedings of the 20th International Conference on Applied Energy (ICAE 2018).

[23] Lin Y.H. A cloud analytics-based electrical energy management architecture empowered by edge analytics AIduino with push notifications for demand-side management.

[24] Zhou B., Li W., Chan K.W., Cao Y., Kuang Y., Liu X., Wang X. (2016). Smart home energy management systems: Concept, configurations, and scheduling strategies. Renew. Sustain. Energy Rev..

[25] Kuzlu M., Pipattanasomporn M., Rahman S. (2014). Communication network requirements for major smart grid applications in HAN, NAN and WAN. Comput. Netw..

[26] Ye F., Qian Y., Hu R.Q. (2015). Energy efficient self-sustaining wireless neighborhood area network design for smart grid. IEEE Trans. Smart Grid.

[27] Haider H.T., See O.H., Elmenreich W. (2016). A review of residential demand response of smart grid. Renew. Sustain. Energy Rev..

[28] Kabalci Y. (2016). A survey on smart metering and smart grid communication. Renew. Sustain. Energy Rev..

[29] Siano P. (2014). Demand response and smart grids—A survey. Renew. Sustain. Energy Rev..

[30] Huang F.L., Tseng S.Y. Predictable smart home system integrated with heterogeneous network and cloud computing. In Proceeding of the International Conference on Machine Learning and Cybernetics (ICMLC).

[31] Hernández-Rojas D.L., Fernández-Caramés T.M., Fraga-Lamas P., Escudero C.J. (2018). Design and practical evaluation of a family of lightweight protocols for heterogeneous sensing through BLE Beacons in IoT telemetry applications. Sensors.

[32] EL Jaouhari S., Jose Palacios-Garcia E., Anvari-Moghaddam A., Bouabdallah A. (2019). Integrated management of energy, wellbeing and health in the next generation of smart homes. Sensors.

[33] R The R Project for Statistical Computing. https://www.r-project.org/.

[34] Vidal-Pardo A., Pindado S. (2018). Design and development of a 5-Channel Arduino-based data acquisition system (ABDAS) for experimental aerodynamics research. Sensors.

[35] Arduino Home Page. https://www.arduino.cc/.

[36] Arduino LLC Language Reference. http://www.arduino.cc/en/Reference/HomePage.

[37] Mnati M.J., Van den Bossche A., Chisab R.F. (2017). A smart voltage and current monitoring system for three phase inverters using an Android smartphone application. Sensors.

[38] Ramya C.M., Shanmugaraj M., Prabakaran R. Study on ZigBee technology. Proceedings of the 3rd International Conference on Electronics Computer Technology (ICECT).

[39] Ding F., Chen X., He S., Shou G., Zhang Z., Zhou Y. (2019). Evaluation of a Wi-Fi signal based system for freeway traffic states monitoring: An exploratory field test. Sensors.

[40] Kim K., Myung H. (2015). Sensor node for remote monitoring of waterborne disease-causing bacteria. Sensors.

[41] Qivicon Smart Home Alliance. https://www.qivicon.com.

[42] Gao L., Wang Z.X., Zhou J.L., Zhang C. (2016). Design of smart home system based on ZigBee technology and R&D for application. Energy Power Eng..

[43] Ullah I., Kim D. (2018). An optimization scheme for water pump control in smart fish farm with efficient energy consumption. Processes.

[44] Sung W.T., Lin J.S. (2013). Design and implementation of a smart LED lighting system using a self adaptive weighted data fusion algorithm. Sensors.

[45] Lin C.T., Lee C.S.G. (2003). Neural Fuzzy Systems: A Neuro-Fuzzy Synergism to Intelligent Systems.

[46] Cooley J.W., Tukey J.W. (1965). An algorithm for the machine calculation of complex Fourier series. Math. Comput..

[47] Heideman M., Johnson D., Burrus C. (1984). Gauss and the history of the fast Fourier transform. IEEE ASSP Mag..

[48] MathWorks Fast Fourier Transform (FFT). http://se.mathworks.com/help/matlab/math/fast-fourier-transform-fft.html.

[49] Viciana E., Alcayde A., Montoya F.G., Baños R., Arrabal-Campos F.M., Zapata-Sierra A., Manzano-Agugliaro F. (2018). OpenZmeter: An efficient low-cost energy smart meter and power quality analyzer. Sustainability.

[50] Kumar S. (2005). Neural Networks: A Classroom Approach.

[51] CRAN Package ‘Neural’. https://cran.r-project.org/web/packages/neural/neural.pdf.

[52] CRAN Package ‘RSNNS’. https://cran.r-project.org/web/packages/RSNNS/RSNNS.pdf.

[53] RDocumentation RSNNS v0.4-11. https://www.rdocumentation.org/packages/RSNNS/versions/0.4-11/topics/rbf.

[54] Wang L.X. (2005). A Course in Fuzzy Systems and Control.

[55] CRAN Package ‘e1071′. https://cran.r-project.org/web/packages/e1071/e1071.pdf.

[56] MathWorks Fuzzy c-Means Clustering. https://www.mathworks.com/help/fuzzy/fcm.html.

[57] CRAN Package ‘matrixcalc’. https://cran.r-project.org/web/packages/matrixcalc/matrixcalc.pdf.

[58] CRAN Package ‘corpcor’. https://cran.r-project.org/web/packages/corpcor/corpcor.pdf.

[59] The DIY Life Simple Arduino Home Energy Meter. https://www.the-diy-life.com/simple-arduino-home-energy-meter/.

[60] ThingSpeak^TM^ Home Page. https://thingspeak.com/.

